# Efficacy of Immunotherapy Combined with Antiangiogenic Therapy in Treatment-Failure Patients with Advanced Carcinoma Ex Pleomorphic Adenoma of the Submandibular Gland: A Case Report

**DOI:** 10.3390/curroncol29090498

**Published:** 2022-09-01

**Authors:** Huanlan Sa, Yinghui Xu, Xiaobo Ma, Xu Wang, Chao Sun, Shi Qiu, Ye Guo, Zhiguang Yang, Yunpeng Liu, Kewei Ma

**Affiliations:** 1 Cancer Center, The First Hospital of Jilin University, Changchun 130021, China; 2 Pathology Department, The First Hospital of Jilin University, Changchun 130021, China; 3 Department of Thoracic Surgery, The First Hospital of Jilin University, Changchun 130021, China

**Keywords:** carcinoma ex pleomorphic adenoma, submandibular gland, immunotherapy, antiangiogenic therapy

## Abstract

Carcinoma ex pleomorphic adenoma (Ca ex PA) is a rare malignant tumor that arises from a primary or recurrent benign pleomorphic adenoma (PA). Ca ex PA has an aggressive behavior and poor prognosis. To date, there are no standardized therapeutic methods. Herein, we reported a case of a 57-year-old Chinese female with Ca ex PA of the submandibular gland. After surgery, cervical lymph nodes recurred, and multiple distant metastases were detected. During the treatment, she received multiple chemotherapies and radiotherapy but suffered from multidrug resistance and repeated disease progression. Hence, PD-1 inhibitor (sintilimab), in combination with anlotinib, was administered, which resulted in better control of pulmonary metastases compared to the other treatment regimens. This provided an alternative treatment option for Ca ex PA of the submandibular gland patients with failed multiple therapies.

## 1. Introduction

Pleomorphic adenoma (PA) is the most common benign salivary gland tumor. Surgical resection is curative, but long-term untreated PA has the potential of malignant transformation. About 6% of PAs are transformed into carcinoma ex pleomorphic adenoma (Ca ex PA) [1], which is a form of malignancy that arises from a primary or recurrent benign pleomorphic adenoma [2]. It has a prevalence rate of 5.6/100,000 malignancies and an annual incidence of 0.17/million individuals [2]. The primary therapeutic option for resectable Ca ex PA is surgical resection—with or without postoperative radiotherapy. Treatment failure is highly correlated with local recurrence and metastasis; the most common metastasis sites are the neck and lungs. Currently, there is no proven effective standard of care for patients with advanced Ca ex PA. A combination chemotherapy of cisplatin, adriamycin, and cyclophosphamide exhibits antitumor effects in some cases; however, prognostic outcomes remain poor, with survival slightly >1 year [3]. Novel treatment regimens are constantly being developed and tried.

Sintilimab is a human immunoglobulin (Ig) G4 monoclonal antibody that binds to programmed cell death receptor-1 (PD-1) by blocking the interaction between PD-1 and its ligands (PD-L1 and PD-L2), thereby restoring the activation and proliferation of T cells [4]. Several clinical studies evaluating the safety and efficacy of sintilimab in patients with advanced cancers, such as classical Hodgkin’s lymphoma, non-small cell lung cancer (NSCLC), hepatocellular carcinoma, and gastric cancer, have shown promising results [5]. Anlotinib, a novel oral multitarget tyrosine kinase inhibitor, targets the vascular endothelial growth factor receptor (VEGFR), fibroblast growth factor receptor (FGFR), platelet-derived growth factor receptors (PDGFR), and stem cell factor receptor (c-Kit), with dual antitumor angiogenesis and tumor growth inhibitory effects [6,7]. Interestingly, anlotinib has significant clinical responses against NSCLC, soft tissue sarcoma, and renal cell carcinoma [6,7].

Herein, we reported a case of metastatic Ca ex PA responsive to a PD-1 inhibitor (sintilimab) combined with anlotinib after the failure of multiple therapies. This is the first case proving the efficacy of immunotherapy plus antiangiogenic therapy for Ca ex PA treatment. Due to the rarity and histological subtype heterogeneity of this tumor, clinical trials may not be conducted for specific subtypes. Hence, such case reports are clinically significant.

## 2. Case Presentation

**Figure 1:**
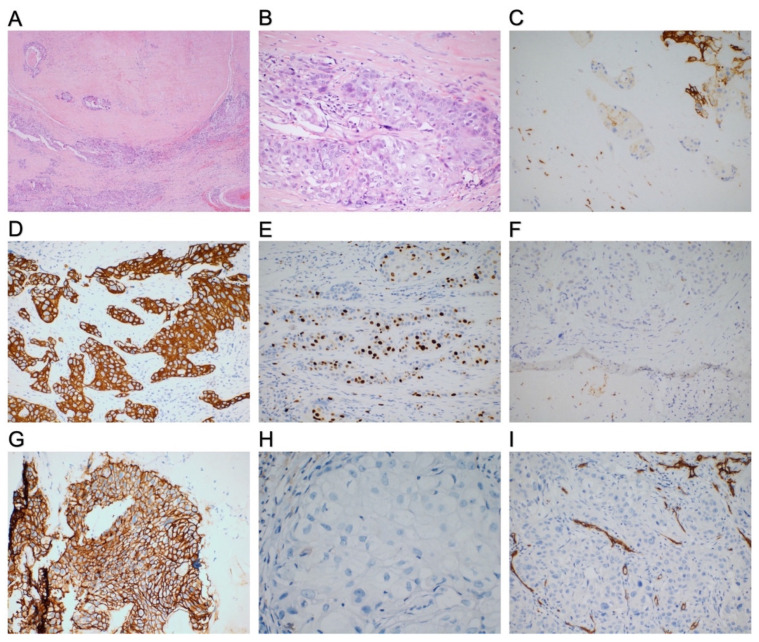
Histological examination of CA ex PA. (**A**) Low magnification showed a PA in the form of a nodule with a fibrous mucinous stroma, with some malignant epithelial components visible within the nodule and at the nodule margin (HE). (**B**) High-power view revealed a solid nest of malignant epithelial components and vesicular nuclei of tumor cells with large, hyperchromatic nuclei and conspicuous nucleoli (HE). (**C**) IHC showed that tumor cells are positive for CK5/6. (**D**) IHC showed that tumor cells are diffuse strong-positive for CK7. (**E**) IHC showed that Ki-67 was about 30%. (**F**) IHC showed that tumor cells were weakly-positive for P63, and the residual myoepithelial cells of peripheral PA were positive. (**G**) IHC showed that tumor cells are strongly positive for the human epidermal growth factor receptor 2 (HER2) (clone 4B5) overexpression. (**H**) IHC showed that tumor proportion score (TPS) for programmed cell death ligand 1 (PD-L1) was <1% (clone 22C3). (**I**) IHC showed that vascular endothelial cells of the stroma are positive for CD34. (A 40×, B–G, and I 200×, H 400× magnification).

A 57-year-old Chinese female with a 2-month history of a painless mass in the left submandibular gland was admitted to the First Hospital of Jilin University in June 2020. Surgery of the left submandibular gland was performed 28 years ago to remove a PA. Computed tomography (CT) combined with a three-dimensional (3D) reconstruction of the neck soft tissue with contrast showed heterogeneous enhancement of the left submandibular gland and revealed a space-occupying lesion. Bilateral carotid sheath and submandibular and submental multiple lymph nodes were also displayed. Chest CT displayed multiple nodules in both lungs, indicating metastasis. Then, resection of the mass in the left submandibular gland, in an en bloc manner and concomitant left neck dissection, was performed. Pathological analyses showed that the carcinoma arose from the PA of the submandibular gland, and the carcinoma component was poorly differentiated as adenocarcinoma (Figure 1A,B). The tumor, 2.8 cm × 2 cm × 2 cm, was invasive, invading about 1 cm outside the envelope. Vasculature and nerves showed cancer infiltrations. The surrounding lymph nodes exhibited tumor infiltration (4/4), while the left cervical lymph node showed tumor metastasis (1/15). Immunohistochemistry (IHC) analysis revealed that the tumor cells were positive for CK5/6, CK7, CK8, CKpan, p63, and Ki-67 (30%), and they were negative for P40, CaIponin, S-100, CD34, estrogen receptor (ER), and progesterone receptor (PR) (Figure 1C–F). In addition, the tumor cells were positive (score 3+) for (human epidermal growth factor receptor) HER2/neu (clone 4B5) (Figure 1G). IHC also showed that the tumor proportion score (TPS) for PD-L1 (clone 22C3) was <1% (Figure 1H), and the combined positivity score (CPS) for PD-L1 (clone 22C3) was <1, while vascular endothelial stromal cells were positive for CD34 (Figure 1I). Pathologically, the final diagnosis was high-grade Ca ex PA. The final stage was T3N2Mx.

Two months after the operation, CT revealed a recurrence of cervical lymph nodes, increased number and size of bilateral pulmonary nodules, and metastasis in the 5th thoracic vertebra. A chemotherapeutic regimen of nab-paclitaxel (370 mg IV day 1) and carboplatin (400 mg IV day 1) was administered. Subsequently, chest CT did not detect any significant changes in bilateral pulmonary metastases. After two and four cycles of the chemotherapeutic regimens, the efficacy was determined as stable disease (SD). Two months later, thoracic spine magnetic resonance imaging (MRI) and radionuclide bone scan revealed multiple bone metastases in the thoracic and lumbar spine and pathological fractures of the 5th thoracic vertebra. Hence, radiotherapy (30 Gy) was administered. On 8 March 2021, the patient developed a swelling in the left mandible, accompanied by numbness in the face. Brain MRI and neck soft tissue CT scans showed multiple metastases in the skull. Chest CT showed enlarged bilateral lung and vertebral metastases. One cycle of cyclophosphamide (800 mg IV day 1), combined with epirubicin (100 mg IV day 1), was administered to the patient. However, due to intolerable vomiting and agranulocytosis as a result of chemotherapeutic-associated toxicities, the treatment was discontinued. Then, skull metastases were subjected to radiotherapy (60 Gy). Brain MRI revealed new metastatic lesions. The rake area was redrawn while skull metastases (42 Gy) and new brain metastasis (52.5 Gy) were subjected to radiotherapy. Consequently, the patient’s facial numbness was relieved.

**Figure 2:**
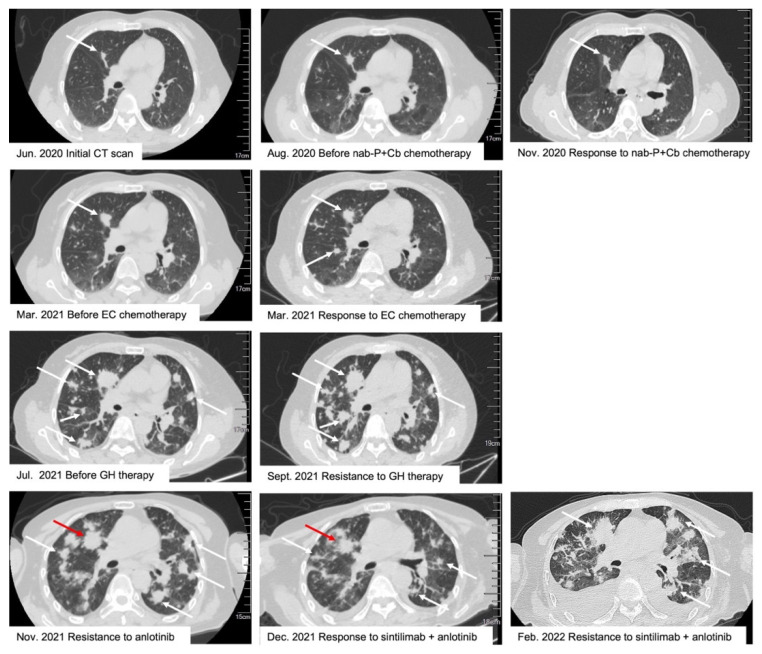
Computed tomography scan images of the patient’s clinical course. Abbreviation: nab-P: nab-paclitaxel; Cb: carboplatin; EC: epirubicin/cyclophosphamide; GH: gemcitabine/trastuzumab. (The red arrow shows the location of the target lesion. The white arrow shows the location of the non-target lesion).

In July 2021, a repeat brain MRI revealed enlarged skull metastases and new lesions. The patient was administered gemcitabine (1.4 g IV day 1,8) plus trastuzumab (390 mg IV every 21 days). After two cycles, the CT scan showed enlarged pulmonary metastases, increased bone metastases, and new liver metastatic lesions. This patient was adjusted to oral anlotinib (12 mg/day on days 1–14, 7 days off, 21-day cycle). After 50 days, chest CT scans showed that pulmonary metastases had increased in number and size. Thus, a combination of sintilimab (200 mg IV every 21 days) and anlotinib was administered. After two cycles, the physical conditions of the patient had improved significantly. Chest CT showed that lung metastases had shrunk (Figure 2). The longest diameter of the target lesion of lung metastasis, before and after two courses of sintilimab in combination with anlotinib, was 24 mm and 18 mm (Figure 2), respectively, and the target lesion was reduced by 25%, and the efficacy was evaluated as SD, according to Response Evaluation Criteria in Solid Tumors version 1.1. Therefore, sintilimab combined with anlotinib was effective for this patient and was not associated with any significant clinical or adverse biological effects. The patient was not followed up in our department, and we found her chest CT that was reviewed on 17 February 2022 (Figure 2). Unfortunately, the chest CT scan suggested enlarged lung metastases and along with pleural effusion, suggesting disease progression with a progression-free survival of 3.5 months. This case suggests that immunotherapy combined with antiangiogenic therapy was effective for this patient in the short term, but we need to accumulate more cases to verify the effectiveness of this combination regimen. Figure 2 shows chest CT scan images of the patient in the clinical process.

## 3. Discussion

In this study, we reported a patient with Ca ex PA of the submandibular gland with cervical lymph node recurrence and multiple distant metastases, with a poor response to multiple therapies. Only sintilimab combined with anlotinib improved the therapeutic outcomes. Both CD31 and CD34 are vascular endothelial markers, but compared with CD31, CD34 is an ideal endothelial marker, which can stain not only normal blood vessels within tumor tissues but also neovascularization [8]. Meanwhile, CD34 has better specificity since CD31 is expressed not only in endothelial cells, but also in platelets and T cells [9]. Soares et al. [8] assessed tumor vascularization in a series of Ca ex PAs representing different phases of the adenoma-carcinoma sequence, i.e., in situ (intracapsular), minimally invasive, and invasive carcinoma, in histological samples by measuring total microvascular area and microvascular density using CD34 antibodies. Soares et al. [8] reported a gradual but significant increase in angiogenesis during the transformation from PA to the extensively aggressive Ca ex PA. Fonseca et al. [10] found that VEGF expression was significantly high in malignant salivary gland tumors compared to benign tumors, suggesting that VEGF is involved in the pathogenesis and aggression of salivary gland tumors. These findings implied that inhibition of the VEGF signaling pathway is a potential treatment approach for Ca ex PA. Song et al. [11] reported a patient with advanced Ca ex PA who achieved partial response after anlotinib monotherapy. However, in the current case, the patient suffered disease progression after 50 days of single-agent anlotinib treatment.

According to the 2017 WHO classification of head and neck cancers, salivary gland carcinomas (SGC), are classified into more than 20 histological tumor subtypes (Table S1), with adenoid cystic carcinoma, mucinous epidermoid carcinoma, acinar cell carcinoma, salivary ductal carcinoma, and adenocarcinoma, not otherwise specified being the most common histological subtypes. The high heterogeneity of biological features and clinical characteristics between subtypes makes standardization of treatment a challenge. Surgical resection is the standard of care for resectable SGCs. Radiotherapy is usually used as adjuvant therapy for tumors with high-risk factors. There is still no established standard of care for advanced SGCs. With the development of molecular biology, immunotherapy and antiangiogenic therapy have been included in the treatment guidelines for a variety of tumors. Investigators are also progressively exploring their efficacy in different histological subtypes of SGCs. We summarized data from a subset of clinical studies (Table S1), with the ORRs for both targeted antiangiogenic drugs (e.g., sorafenib, axitinib, and lenvatinib) and immunotherapy, ranging from 0 to 16% [12,13,14,15,16,17,18,19,20]. There is an ongoing phase II clinical study of immunotherapy combined with anti-angiogenesis for SGCs (ClinicalTrials.gov, NCT04209660) [21]. We look forward to the release of the trial results.

The overexpression of PD-L1 in the SGC tumor cells promoted immune tolerance and progression in SGCs [22,23]. Harada et al. [23] found a significant correlation between PD-L1 expression in SGC tumor cells with metastatic recurrence, after surgery and survival, in SGC patients [23]. Recent studies have shown that PD-1 inhibitors (pembrolizumab and nivolumab) alone or in combination with vorinostat have limited efficacy in the treatment of progressive SGC [12,13,15]. However, no cases of Ca ex PA were reported in these studies.

A combination of immunotherapy and antiangiogenic therapy can block the PD-1/PD-L1 axis to synergistically inhibit tumor growth, especially in tumors with high secretions of VEGF. Moreover, antiangiogenic therapy can normalize tumor blood vessels and reshape the tumor immune microenvironment [24]. Some studies have reported PD-L1 expression levels in SGCs, but most of them have focused on the prognostic value of PD-L1 expression [23,25]. Up to now, no PD-L1 expression scoring standard has been established in SGCs to evaluate the efficacy of immune checkpoint inhibitors therapy, and even whether PD-L1 can be used as a predictor of efficacy of immune checkpoint inhibitors therapy for SGCs is unclear [25]. Witte et al. [25] tried to use the scoring criteria established by TPS or CPS cutoff values determined in NSCLC (KEYNOTE-042) [26] or gastric cancer (KEYNOTE-059) [27] to evaluate the expression of PD-L1 in SGCs and whether it could be used as a target to predict the efficacy of immunotherapy. However, due to the limited sample size of patients receiving immunotherapy, the efficacy of immunotherapy for SGCs cannot be further statistically inferred [25]. IMpower150 [28] is a phase III trial of immunotherapy combined with anti-angiogenesis therapy for advanced non-squamous NSCLC. The data published in this study show that, regardless of the expression level of PD-L1, antiangiogenic drugs combined with immunotherapy can bring significant progression-free survival and overall survival benefits to patients. The patient was already in a situation where multiple chemotherapy regimens, targeted therapy, and radiation therapy had failed in multiple lines of treatment. Therefore, based on this consideration, we expect that this patient would also benefit from the combination of immune checkpoint inhibitors with antiangiogenic drugs for survival. In advanced NSCLC [29] and cervical cancer [30], sintilimab, in combination with anlotinib, exerts synergistic antitumor effects. However, treatment of advanced Ca ex PA with immunotherapy plus antiangiogenic therapy is not yet reported. To the best of our knowledge, this is the first report on the combination of PD-1 inhibitors and antiangiogenic drugs for advanced Ca ex PA treatment. After multiline therapy failure, sintilimab, combined with anlotinib, showed optimal clinical efficacy.

PD-L1 is a biomarker for various tumor immunotherapies. However, several studies have reported different PD-L1 levels in SGCs [31]. Therefore, identifying appropriate biomarkers and developing rational treatment regimens would be clinically significant. Lymphocyte activation gene 3 (*LAG3*) is overexpressed in most SGCs [32]. The combined blockade of LAG3 and PD-1 can restore the antitumor activities [33]. Haghshenas et al. demonstrated a critical role of chemokines and chemokine receptors in the invasive behavior of SGCs and, thus, may be candidate targets for cancer immunotherapy [31].

## 4. Conclusions

This is the first report using a combination of immunotherapy and antiangiogenic therapy for advanced Ca ex PA of the submandibular gland. The current findings render that immunotherapy, combined with antiangiogenic therapy, is promising as an alternative treatment option for Ca ex PA. Nonetheless, additional studies are required to verify these findings, and it is crucial to explore appropriate immunotherapeutic biomarkers for optimal beneficiaries.

## Data Availability

The data presented in this study are available on request from the corresponding author.

## Supplementary Materials

The following supporting information can be downloaded at: https://www.mdpi.com/article/10.3390/curroncol29090498/s1, Table S1: Summary of histological types of salivary gland cancers (SGCs) and advances in immunotherapy and antiangiogenic therapy.
