# Supplementary material for: Efficacy of Immunotherapy Combined with Antiangiogenic Therapy in Treatment-Failure Patients with Advanced Carcinoma Ex Pleomorphic Adenoma of the Submandibular Gland: A Case Report
Source: Curr Oncol. 2022 Sep 1;29(9):6334–41. doi: 10.3390/curroncol29090498 (PMC9497983; doi:10.3390/curroncol29090498)
Supplement: Supplementary file 1 [file curroncol-29-00498-s001.zip › curroncol-1806051-supplementary.pdf]

Table S1 Summary of histological types of salivary gland cancers (SGCs) and advances in immunotherapy and antiangiogenic therapy

Histological Subtypes *	Current treatment principle	Study	Histological subtypes	Therapy	ORR (%)
Mucoepidermoid carcinoma (MEC)	①For resectable SGCs, the standard of care is surgical resection with or without postoperative radiotherapy.<br>②For advanced SGCs, there is no proven standard of care.	Immunotherapy			
Adenoid cystic carcinoma (ACC)<br>Acinic cell carcinoma (AcCC)<br>Polymorphous adenocarcinoma		Niwa et al. [12]	SDC, Adenocarcinoma, ACC, MEC	Nivolumab	4.2
Clear cell carcinoma (CCC)<br>Basal cell adenocarcinoma<br>Intraductal carcinoma<br>Adenocarcinoma, not otherwise specified<br>Salivary duct carcinoma (SDC)		Cohen et al. [13]	Adenocarcinoma, MEC, Undifferentiated carcinoma, SCC, ACC	Pembrolizumab	12
Myoepithelial carcinoma (MECA)<br>Epithelial-myoepithelial carcinoma (EMC)		Mahmood et al. [14]	ACC	Pembrolizumab vs Pembrolizumab + RT	0
Carcinoma ex pleomorphic adenoma (Ca ex PA)<br>Secretory carcinoma<br>Sebaceous carcinoma		Rodriguez et al. [15]	ACC, AcCC, MEC, Adenocarcinoma, LEC	Pembrolizumab + Vorinostat	16
Carcinosarcoma		Antiangiogenic therapy			
Poorly differentiated carcinoma		Tchekmedyian et al. [16]	ACC	Lenvatinib	15.6
Undifferentiated carcinoma<br>Large cell neuroendocrine carcinoma<br>Small cell neuroendocrine carcinoma<br>Lymphoepithelial carcinoma (LEC)<br>Squamous cell carcinoma<br>Oncocytic carcinoma		Locati et al. [17]	ACC, Adenocarcinoma, Poorly differentiated carcinoma, AcCC, CCC	Axitinib	8

Uncertain malignant potential<br>Sialoblastoma	Locati et al. [18]	ACC, MEC, Adenocarcinomas, SDC, Poorly differentiated carcinoma, MECA	Sorafenib	16
	Chau et al. [19]	ACC	Sunitinib	0
	Keam et al. [20]	ACC	Dovitinib	3.1
	Immunetherapy plus antiangiogenic therapy			
	NCT04209660[21]	ACC and Other SGCs	Lenvatinib + Pembrolizumab	NR

Abbreviation: ORR: objective response rate; NR: Not Reported. *Histological classification of Salivary gland cancers according to the 2017 WHO classification of head and neck cancers.
